# Improving Staff-Family End-of-Life Communication at Israeli Geriatric Facilities by Using a Mobile App

**DOI:** 10.1093/geroni/igab046.1992

**Published:** 2021-12-17

**Authors:** Rinat Cohen, Gal Maydan, Shai Brill, Jiska Cohen-Mansfield

**Affiliations:** 1 Tel-Aviv University, Hadera, Hefa, Israel; 2 Beit Rivka, Petah-Tikva, HaMerkaz, Israel; 3 Clalit Health Services, Tel-Aviv, Tel Aviv, Israel; 4 Tel-Aviv University, Tel-Aviv, Tel Aviv, Israel

## Abstract

Family caregivers (FCs) of persons institutionalized at geriatric facilities present significant unmet communication needs regarding receiving regular updates about their loved one’s condition and having available healthcare professionals (HPs) to approach when needed. We developed and tested a mobile-app for staff-family communication with both parties having active roles in app planning to tailor it to their needs and abilities. The app includes a daily-update module for FCs and a chat option for FCs and HPs. App use was piloted at one geriatric-medical-center for 15 months (unit-level randomization resulted in one complex-care and one assisted-ventilation unit in each group- intervention and control) and one single-unit nursing-home for three months. Personal interviews were conducted with 55 FCs (28 from intervention-group and 27 FCs from control-group) before-and-after app use (with mean duration of use 1.6[S.D.=.6] months. Most participants were women and the children of the patients; their mean age was 55.9 years (S.D.=12.4). Repeated-measures Analysis-of-Variance for the end-of-life communication sub-scale on the Quality-of-communication questionnaire yielded a main effect for time (F(1,53)=8.31, p=.006) with both groups’ ratings increasing over time and an interaction effect (F(1,53)=4.78, p=.033) with a greater increase for intervention-group compared to control-group. Intervention-group participants rated the app as convenient to use. Qualitative data revealed that FCs perceived app use as improving quality of communication with the HPs who used it and improving their own well-being. The app offers a feasible and an effective mode of communication that incorporates technology in daily communication between FCs and HPs while addressing FCs’ unmet needs.

